# Apoptosis induction associated with the ER stress response through up-regulation of JNK in HeLa cells by gambogic acid

**DOI:** 10.1186/s12906-015-0544-4

**Published:** 2015-02-15

**Authors:** Aungkana Krajarng, Masaya Imoto, Etsu Tashiro, Takahiro Fujimaki, Satoko Shinjo, Ramida Watanapokasin

**Affiliations:** Chulabhorn International College of Medicine, Thammasart University, Pratumthani, Thailand; Department of Biochemistry, Faculty of Medicine, Srinakharinwirot University, Bangkok, Thailand; Department of Biosciences and Informatics, Faculty of Science and Technology, Keio University, Yokohama, Japan

**Keywords:** Gambogic acid, Anticancer, ER stress, Apoptosis, HeLa cells

## Abstract

**Background:**

Gambogic acid (GA) was extracted from the dried yellow resin of gamboge (*Garcinia hanburyi*) which is traditionally used as a coloring material for painting and cloth dying. Gamboge has been also used as a folk medicine for an internal purgative and externally infected wound. We focused on the mechanisms of apoptosis induction by GA through the unfold protein response (ER stress) in HeLa cells.

**Methods:**

The cytotoxic effect of GA against HeLa cells was determined by trypan blue exclusion assay. Markers of ER stress such as XBP-1, GRP78, CHOP, GADD34 and ERdj4 were analyzed by RT-PCR and Real-time RT-PCR. Cell morphological changes and apoptotic proteins were performed by Hoechst33342 staining and Western blotting technique.

**Results:**

Our results indicated a time- and dose-dependent decrease of cell viability by GA. The ER stress induction is determined by the up-regulation of spliced XBP1 mRNA and activated GRP78, CHOP, GADD34 and ERdj4 expression. GA also induced cell morphological changes such as nuclear condensation, membrane blebbing and apoptotic body in Hela cells. Apoptosis cell death detected by increased DR5, caspase-8, −9, and −3 expression as well as increased cleaved-PARP, while decreased Bcl-2 upon GA treatment. In addition, phosphorylated JNK was up-regulated but phosphorylated ERK was down-regulated after exposure to GA.

**Conclusions:**

These results suggest that GA induce apoptosis associated with the ER stress response through up-regulation of p-JNK and down-regulation of p-ERK in HeLa cells.

## Background

Gambogic acid (GA) is the major active ingredient of gamboge extracted from *Garcinia hanburyi* in Southeast Asia, India and China. It is commonly known in Thailand as “Rong Thong”. It has been used as a folk medicine for an internal purgative and externally infected wound [1]. GA has potent anticancer effect, including induction of apoptosis and cell cycle arrest in a broad range of human cancer such as gastric cancer [2], leukemia [3], breast cancer [4] and hepatoma cancer [5]. It has been demonstrated that GA activates cell apoptosis through transferrin receptor [6] and nuclear factor-κB signaling pathway [7]. Previous study also showed that GA has anti-metastasis activity by suppressing the expression of integrin β1 [8]. Moreover, GA could inhibit tumor angiogenesis by decreasing VEGF production [9] and suppressing VEGFR2 [10]. Recently, Zhang et al. [11] showed that GA could be an inhibitor of Heat shock protein 90 (Hsp90) which is a key protein in cell signaling and tumor regeneration. Inhibition of Hsp90 by treatments of cells with specific inhibitors leads to ER stress induction and subsequent activation of the three ER stress sensors of the unfolded protein response which contributed to cell death of the treated cells [12].

The endoplasmic reticulum (ER) is a central organelle involved in lipid synthesis, protein folding and maturation. The ER is highly sensitive to stress that disturb cellular energy levels, the redox state or Ca^2+^ concentration. These reduce the protein folding capacity of the ER and cause the accumulation and aggregation of unfolded protein which result in ER stress response termed unfolded protein response (UPR). The ER stress response is regulated by three ER transmembrane receptors; pancreatic ER kinase (PKR)-like ER kinase (PERK), inositol requiring enzyme 1 (IRE1) and activating transcription factor 6 (ATF6) [13]. These ER transmembrane proteins are kept in an inactive state through their association with the ER chaperone BiP/GRP78 (glucose-related protein, 78kD). During ER stress, GRP78 dissociates from these three transmembrane proteins. Activated PERK blocks general protein synthesis by phosphorylating eukaryotic initiation factor 2 (eIF2α). This phosphorylation enables translation of ATF4 which translocates to the nucleus and induces the transcription of genes required to restore ER homeostasis. Activation of IRE1 by ER stress is typical of receptor kinase proteins, which homodimerize and transphosphorylate [14]. IRE1 splices X-box protein 1 (XBP1) mRNA to form mature XBP1s mRNA (s for spliced) which leads to not only the transcriptional activation of ER-associated protein degradation (ERAD) component genes and ER/Golgi biogenesis but also genes involved in redox homeostasis and oxidative stress response [15,16]. After the dissociation of GRP78, ATF6 translocates to the Golgi apparatus where it is cleaved into its active form by site-1 and site-2 protease. Active ATF6 then binds to ER stress response element (ERSE) in the nucleus to activate transcription of ER chaperone genes such as GRP78, GRP94, and the transcription factors C/EBP homologous protein (CHOP) and XBP1 [17]. ER stress has recently been identified as another major pathway engaged in the initiation of apoptosis [18]. Severe or prolonged ER stress stimulates PERK, ATF6 and IRE1 apoptotic signaling and increase CHOP expression. It has been showed that CHOP is a critical ER stress-induced apoptosis molecule through regulating the expression of Bcl2, Bim and DR5 [19,20].

Cervical cancer is the fourth most common cancer in woman worldwide and it remains a leading cause of death from cancer in developing and low income countries [21]. In this study, we have shown for the first time that GA can induce apoptosis in cervical cancer HeLa cells associated with the ER stress response by up-regulation of CHOP, p-JNK and down-regulation of p-ERK.

## Methods

### Preparation of gambogic acid (GA)

**Figure 1:**
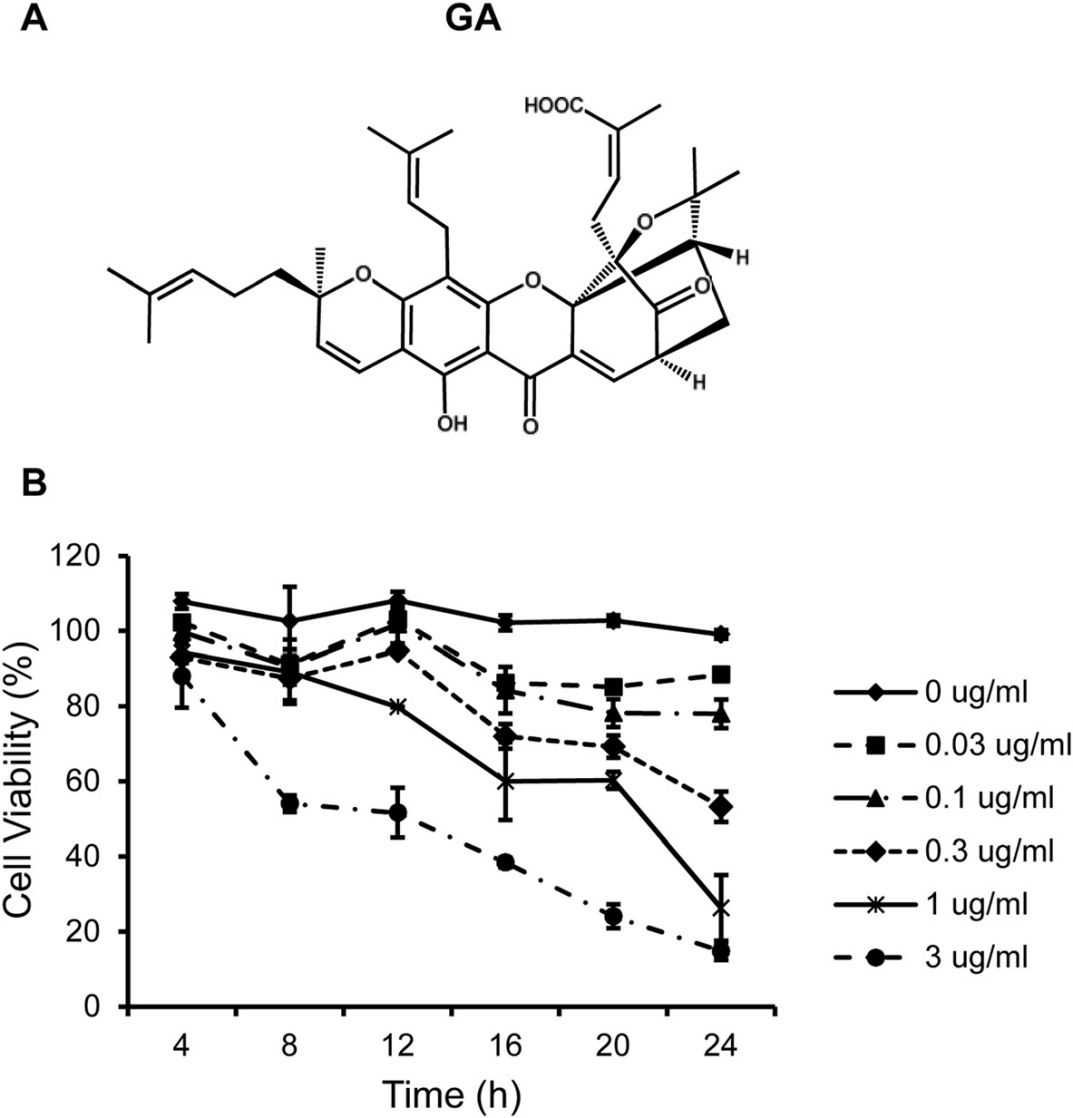
**GA inhibits cell growth in HeLa cells. (A)** Formula of Gambogic acid (GA). **(B)** Effect of GA on cell viability in HeLa cells. Time- and dose-dependent effect of GA was performed when HeLa cells were treated with various concentrations of GA at different time points and their viability was determined by trypan blue exclusion assay. Results are mean values ± SD of three independent experiments (n = 3).

*Garcinia hanburyi* was collected from Laem-ngob, Trat province, Thailand in Jan 2012. A voucher specimen (Montree Kurukitkoson No. 001) was deposited at the Faculty of Medicine, Srinakharinwirot University, Bangkok, Thailand. GA (Figure 1A) was isolated from gamboge (*G. Hanburyi*). The extraction and separation method was as followed: dried resin of gamboge (1 g) was grounded into a powder and extracted with acetone: dH_2_0 (1:1, 1 L), followed by ethyl acetate (1:1). After evaporation, the extract yielded as a yellowish solid (0.6 g). A portion of extract (0.3 g) was subjected to silica gel column chromatography (Silica gel 60, 60–230 μm, Merck, Darmstadt, Germany) using chloroform/methanol stepwise system and yielded the major compound, GA, including other minor compounds. Repeated purification of these column fractions was performed on a SSC-1311 recycling HPLC system equipped with a SSC-5410 UV–vis detector and SSC-3462 pump (Senshu Scientific, Japan). The column was Capcell Pak C18, type UG80 (20 mm id. × 250 mm, 5 μm, Shiseido, Japan). The mobile phase was 75% acetonitrile at a flow rate of 10 ml/min. The UV detection wavelength was set at 360 nm. The chemical structures of GA were then identified by comparing their ^1^H NMR and ^13^C NMR spectra (JNM-ECA500 NMR, JEOL, Japan) with the literature data [22]. GA was dissolved and diluted in methanol (Wako, Japan) at the desired concentration for assays.

### Cell culture

Human cervical carcinoma HeLa cells were obtained from the American Type Culture Collection (ATCC, Manassas, VA). Cells were cultured in Dulbecco’s modified Eagle’s medium (Nissui, Tokyo, Japan) containing 8% fetal bovine serum, 2.5 g/L sodium bicarbonate, and penicillin G (100 units/mL).The medium was refreshed every 2–3 days. After about 70% confluence, the cultured cells were detached with 0.25% trypsin-EDTA and sub-cultured.

### Cell viability

The effect of GA on cell viability was analyzed by using trypan blue exclusion method. Cells were seeded in a 48-well plate at 1 × 10^4^ cells/well and incubated overnight. Then, the cells were treated with the indicated concentrations (0, 0.03, 0.1, 0.3, 1, 3 μg/mL) of GA for 4, 8, 12, 16, 20 and 24 h. The control cells were treated with the solvent (methanol) used to prepare the GA. Cells were trypsinized and stained with trypan blue then counted with a hemocytometer. Cell survival was expressed as percentage of viable cells to total cells. Cells were treated in triplicate, and the experiments were repeated three times.

### RT-PCR analysis

HeLa cells were seeded in a 12-well plate at 5 × 10^4^ cells/well for overnight. Then cells were treated with 0.3 μg/mL of GA, 10 μg/mL tunicamycin as a positive control, for 4 and 8 h. Then total RNA from HeLa cells was extracted by using TRIzol reagent (Invitrogen, Carlsbad, CA). The cDNA was reverse transcribed from 2 μg of total RNA with oligo (dT) and M-MLV reverse transcriptase (Promega, Madison, WI). PCR was carried out with KOD Plus polymerase (Toyobo, Osaka, Japan) using a pair of primers corresponding to nucleotides 505–525 (AATGAAGTGAGGCCAGTGGCC) and 609–629 (CCCATGGATTCTGGCGGTATT) of XBP1 cDNA. The amplified products were separated by electrophoresis on 8% polyacrylamide gel and visualized with ethidium bromide staining.

### Real-time RT-PCR

For quantification, real-time PCR was performed with SYBR Premix Ex Taq (Takara, Siga, Japan). The primers used for amplification were as followed: GRP78, forward 5′-GCTCGACTCGAATTCCAAAG-3′ and reverse 5′-GATCACCAGAGAGCACACCA-3′; CHOP, forward 5′-GCGCATGAAGGAGAAAGAAC-3′ and reverse 5′-TCACCATTCGGTCAATCAGA-3′; ERdj4, forward 5′-AAAATAAGAGCCCGGATGCT-3′ and reverse 5′-CGCTTCTTGGATCCAGTGTT-3′; GADD34, forward 5′-AAACCAGCAGTTCCCTTCCT-3′ and reverse 5′-CTCTTCCTCGGCTTTCTCCT-3′ and GAPDH, forward 5′-AGGTCGGAGTCAACGGATTT-3′ and reverse 5′-TAGTTGAGGTCAATGAAGGG-3′

### Nuclear morphology staining with Hoechst 33342

HeLa cells (2 × 10^5^ cells/well in a six-well plate) were treated with 0.3 μg/mL GA for 0, 8, 16 and 24 h. After trypsinization, cells were washed with 1X PBS and stained with 3 μg/ml of Hoechst 33342 (Molecular Probes, Invitrogen, USA) for 15 min. Stained cells were examined using fluorescence microscope (Olympus, Tokyo, Japan) with an ultraviolet filter.

### Western blot analysis

HeLa cells were incubated for different times in the presence or absence of 0.3 μg/mL of GA, harvested and washed once with ice cold PBS. Then, 2 × 10^5^ cells were lysed for 30 min on ice in 50 μl of RIPA lysis buffer (50 mM Tris–HCl, pH 7.5, 5 mM EDTA, 250 mM NaCl, 0.5% Triton X-100) containing complete mini protease inhibitor cocktail (Roche Diagnostics GmbH, Mannheim, Germany). Clear cell lysate supernatants were prepared by centrifugation and the protein content was determined using Bio-Rad protein assay (Bio-Rad Laboratories, USA). Proteins were separated by 12% SDS-PAGE and transferred onto polyvinylidene fluoride (PVDF) membranes (Pall Corporation, USA) for 1 h at 100 V with the use of a Mini Trans-Blot Cell® (Bio-Rad). After blocking with TBST (10 mM Tris, pH 7.5, 150 mM NaCl and 0.1% Tween 20) containing 5% nonfat milk, the blots were incubated overnight at 4°C with primary antibody (Cell Signaling Technology, Beverly, MA.) The membranes were washed in TBST and the appropriate secondary antibody conjugated with horseradish peroxidase (Cell Signaling Technology, Beverly, MA) was added for 1 h at room temperature. Immunoreactive protein bands were detected by chemiluminescence using enhanced chemiluminescence reagent (ECL, Millipore, Bedford, MA). The membranes were stripped and reprobed with β-actin antibody to assess protein loading for each lane.

### Statistical analysis

All values were represented as mean ± S.D. One way analysis variance (ANOVA) was used in multi comparisons between groups. Statistical significance is accepted at *p* < 0.05.

## Results

### GA decreases cell viability in HeLa cells

We first examined the effect of GA on the cell viability of HeLa cells. The cells were treated with various concentrations of GA (0–3 μg/ml) for 4, 8, 12, 16, 20, 24 h. The cell viability was then evaluated using trypan blue exclusion method. The result showed that GA decreased cell viability in a time- and dose-dependent manner (Figure 1B). GA concentration at 0.3 μg/ml was used for subsequent experiments.

### GA induces ER stress induction in HeLa cells

**Figure 2:**
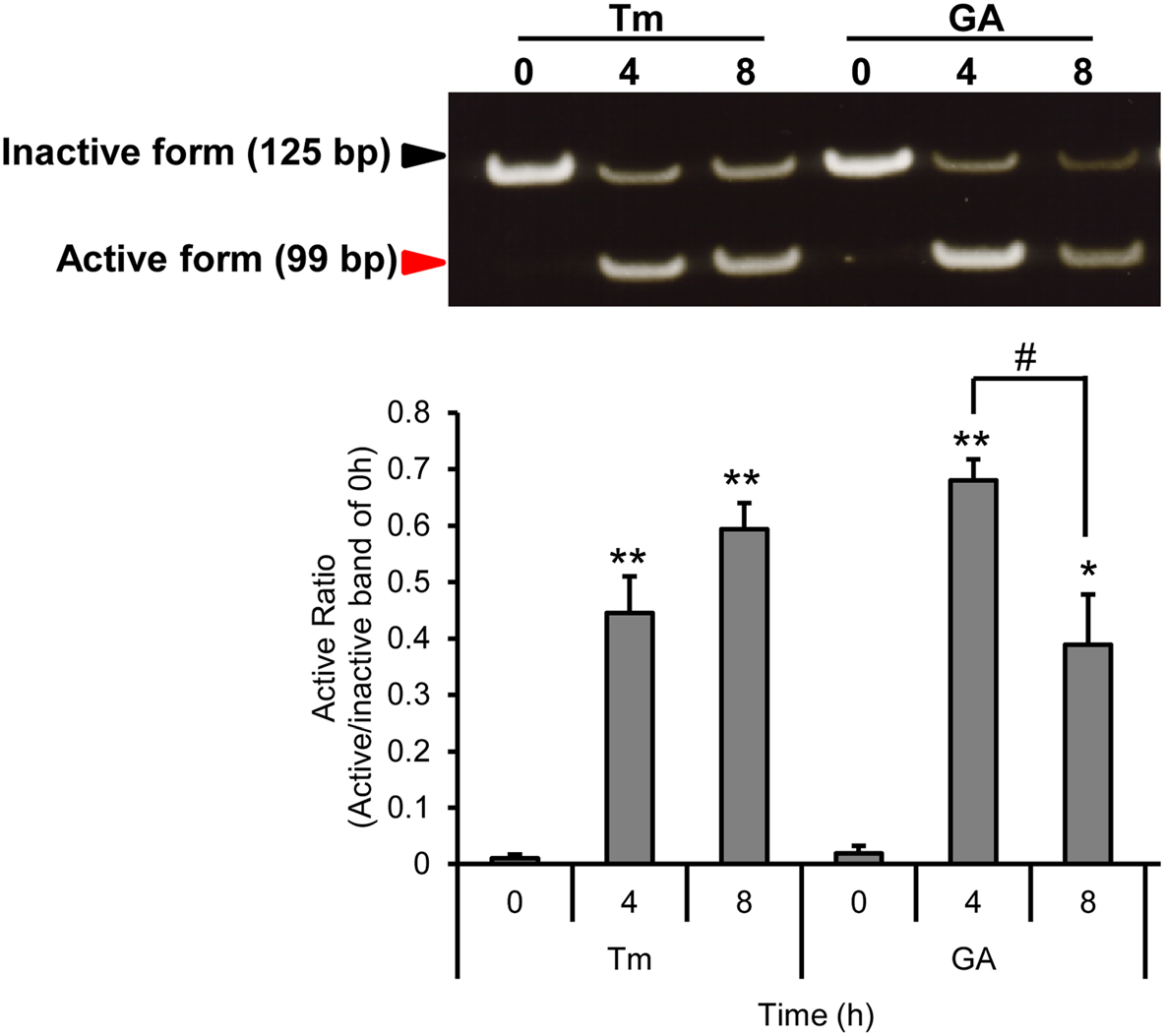
**GA induces XBP1 mRNA splicing in HeLa cells.** HeLa cells were treated with 10 μg/ml of tunicamycin (Tm) or 0.3 μg/ml of GA for 0, 4 and 8 h. mRNA was extracted and subjected to the RT-PCR. The active form was normalized to the inactive form at 0 h. Results are mean values ± SD of three independent experiments (n = 3). *p < 0.05; **p < 0.01 shows significant difference compared with 0 h; #p < 0.05, is significantly different as compared between 4 h and 8 h.

In response to ER stress, IRE1 splices a 26 nucleotide long intron of inactive unspliced XBP1 mRNA (XBP1u), generating an active and stable transcription factor XBP1s. To access whether GA triggers ER stress, we analyzed XBP1 mRNA splicing in HeLa cells. The XBP1 cDNA was amplified by RT-PCR. Tunicamycin (Tm), a well-recognized inducer of ER stress, served as a positive control in these tests. The result showed GA induced XBP1 splicing at 4 h and the XBP1 mRNA was elevated by approximately 6 fold compared to the level observed in control cells (0 h) (Figure 2). In contrast to Tm, the expression of XBP1 splicing was decreased following treatment with GA for 4 h to 8 h.

**Figure 3:**
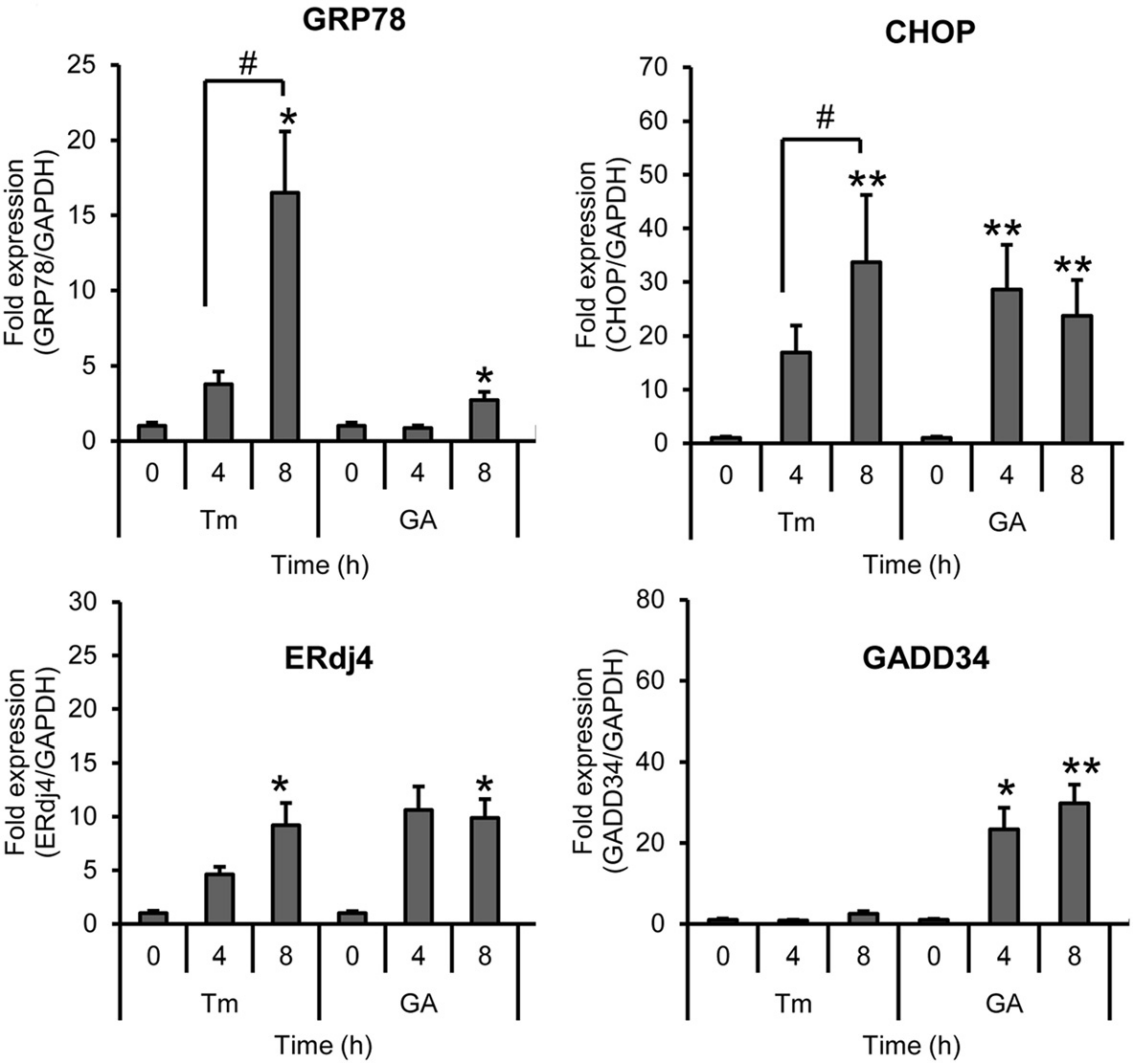
**GA induces ER stress via up-regulation of GRP78, CHOP, ERdj4 and GADD34 mRNA.** HeLa cells were treated with 10 μg/ml of tunicamycin (Tm) or 0.3 μg/ml of GA for 0, 4 and 8 h. Cells were collected and mRNA was extracted and evaluated by real-time RT-PCR. Each mRNA was normalized with the mRNA of GAPDH. Data are the results of three independent experiments, expressed as the mean ± SD, n = 3. *p < 0.05; **p < 0.01 shows significant difference compared with 0 h; #p < 0.05, is significantly different as compared between 4 h and 8 h.

We further examined whether GA induced ER stress. The mRNA expression levels of ER stress-associated molecules (GRP78, CHOP, endoplasmic reticulum-localized DnaJ homologues (ERdj4) and growth arrest and DNA damage-inducible protein (GADD34)) were investigated by real -time PCR. CHOP, also known as GADD153, encodes a member of the CCAAT/enhancer-binding protein family and acts as an inhibitor or activator of transcription, leading to apoptosis [20]. ERdj4, which is a chaperone protein localized in the ER, is a downstream gene of XBP1, while GADD34 is downstream gene of eIF2α. Figure 3 showed GRP78 was slightly increased at 8 h in cells treated with GA. On the other hand, Tm showed obviously increased GRP78 expression and the highest level at 8 h. In addition, there was a large increase in CHOP and ERdj4 level between 4 and 8 h following exposure to Tm and GA. It is noticed that GA increased GADD34 expression, while it had very little effect in cells treated with Tm. All of these observations strongly imply that GA induced ER stress in HeLa cells.

### GA induce apoptosis through intrinsic and extrinsic pathways in HeLa cells

**Figure 4:**
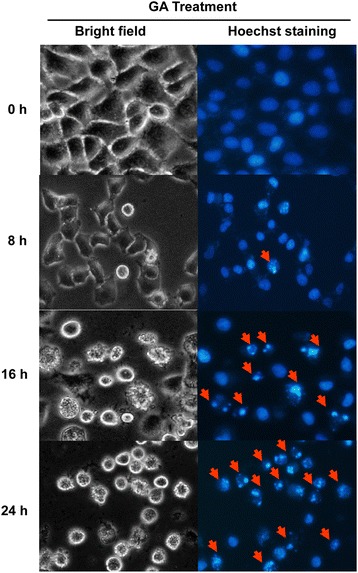
**Effect of GA on cell morphological changes.** HeLa cells were treated with 0.3 μg/ml of GA for 0, 8, 16 and 24 h. Cells were stained with Hoechst33342 and examined under a bright-field and fluorescence microscope (40X magnification). The arrows show cell morphological changes of apoptotic cells.

To observe the morphological effects of HeLa cells in a time-dependent incubation of GA, cells were stained with Hoechst33342 and examined under a bright-field and fluorescence microscope. It showed that GA-treated cells which had nuclear condensation, membrane blebbing, shrunken and became apoptotic body in a time-dependent manner, while the control cells were of round shapes (Figure 4). This result indicated that the morphological changes of HeLa cells by GA were due to apoptosis.

**Figure 5:**
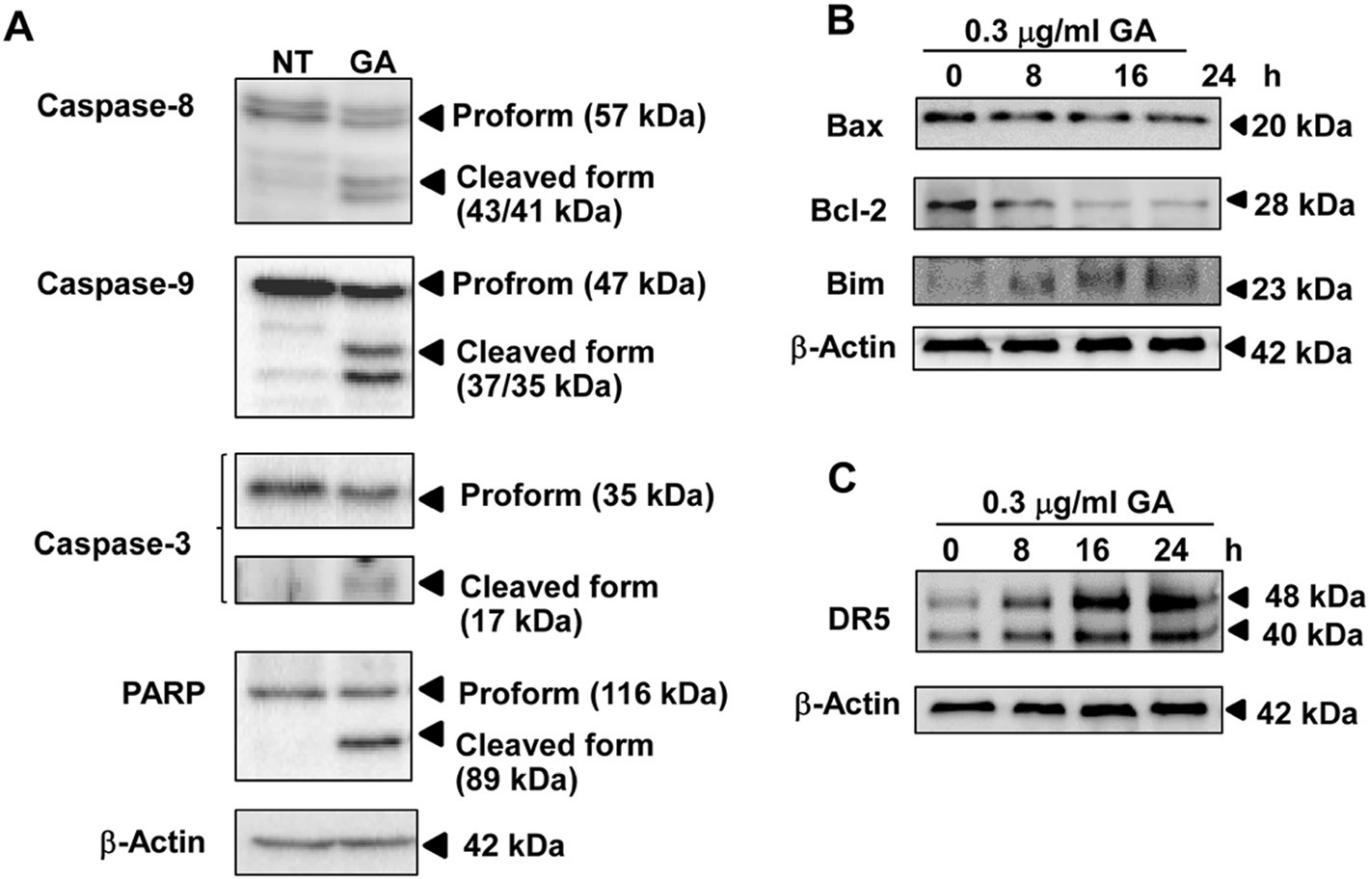
**GA induces apoptosis. (A)** The expression of caspase-3, −8, −9, and PARP were analyzed by Western blotting. HeLa cells were non treated (NT) or treated with 0.3 μg/ml of GA for 24 h. Effect of GA on the expression of **(B)** Bcl-2 family and **(C)** DR5 in HeLa cells. HeLa cells were treated with 0.3 μg/ml of GA for the indicated time points and analyzed by Western blotting. The results from representative experiments were expressed relative to the protein level at 0 h after normalization to β-actin signals.

To examine whether GA can induce apoptosis in HeLa cells, we examined the activation of caspases and Poly (ADP-ribose) polymerase (PARP), a substrate of caspase 3, by Western blotting. As shown in Figure 5A, GA reduced procaspase-8, −9 and −3 protein levels and increased active cleaved caspase-8, −9 and −3 and PARP cleavage. These results demonstrate that GA is involved in apoptosis induction in HeLa cells.

ER stress-induced apoptosis occurs in both intrinsic and extrinsic apoptosis pathways. Bcl-2 family of proteins such as Bcl-2, Bax and Bim are involved in regulating the intrinsic or mitochondrial apoptotic pathway. Western blotting results revealed the decreased Bcl-2 level in GA-treated cells in a time-dependent manner (Figure 5B). No significant change in Bax level was observed upon GA treatment. Previous study reported that Bim, a pro-apoptotic BH3-only member of the Bcl-2 family, is essential for ER stress-induced apoptosis [23]. In this study, GA increased Bim in a time-dependent manner. These results demonstrate that the mitochondrial pathway is involved in GA-induced apoptosis in HeLa cell.

DR5 expression can be regulated at the transcriptional level through CHOP via binding to the CHOP-binding site in the DR5 gene 5′-flanking region [20]. DR5 also activates the downstream caspase-8 and the extrinsic apoptotic pathway [24]. The result showed DR5 protein level was increased in a time-dependent manner following GA treatment (Figure 5C). Our data suggest that DR5-mediated extrinsic apoptotic pathway is involved in GA-induced HeLa cell death.

### Effect of GA on MAPK pathway

**Figure 6:**
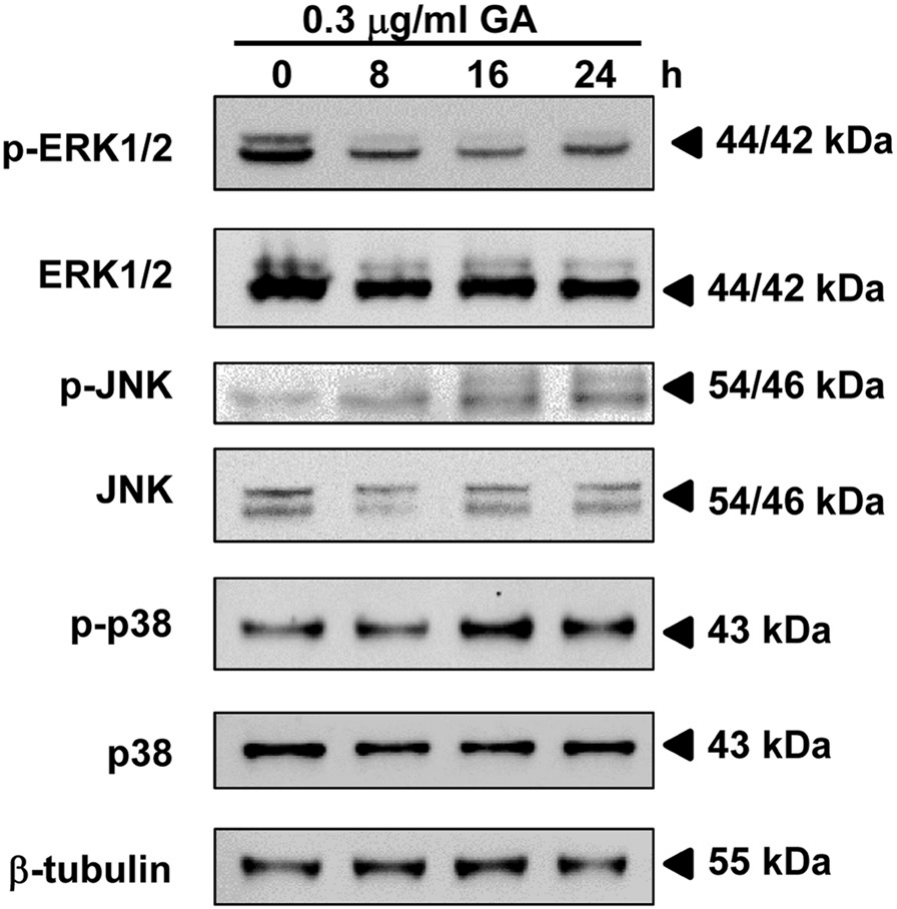
**Effect of GA on MAPK pathway in HeLa cells.** HeLa cells were treated with 0.3 μg/ml of GA for the indicated time points. The expression of phosphorylated and total ERK1/2, JNK and p38 were analyzed by Western blotting and were expressed relative to the protein level at 0 h after normalization to β-actin signals.

MAPKs are important signaling members of serine/threonine protein kinase family that control cellular proliferation, differentiation, survival and apoptosis. Three major mammalian MAPK subfamilies, extracellular regulated protein kinase (ERK), c-Jun N-terminal kinase (JNK) and p38, were activated through a specific phosphorylation cascade. In HeLa cells, GA decreased the level of phosphorylated ERK1/2 but not the total form (Figure 6). On the other hand, GA increased the level of phosphorylated JNK but decreased the level of total JNK protein after treatment with the compounds. In addition, GA did not affect p38 activity. The results suggested that GA may induce ER stress and apoptosis through the ERK and JNK MAPK pathways.

## Discussion

GA, the major active compound extracted from gamboge, has been shown to induce apoptosis and inhibit cell growth in several cancer cell lines. However, this is the first study showing that ER stress is involved in GA-induced apoptosis in HeLa cells through up-regulaion of CHOP and p-JNK. Our study shows that GA can inhibit proliferation of HeLa cells in a dose- and time-dependent manner. GA concentration at 0.3 μg/ml was used for subsequent experiments because it showed the best results of ER stress but did not induce cell death at 4–8 h.

ER plays an important role in the maintenance of intracellular calcium homeostasis, protein synthesis, posttranslational modifications and protein folding. Recently, the potential of ER stress in tumor is considered important for regulating the balance between tumor cell death and growth, and for developing the sensitivity of chemotherapeutic agents [25]. ER stress is regulated by transmembrane proteins; PERK, IRE1 and ATF6. All of the three transmembrane proteins are activated through dissociation with GRP78 when there is an imbalanced of unfolded proteins and chaperones. The activation of IRE1 promotes XBP1 splicing. Spliced XBP1 is an active transcription factor that regulates the transcription of many target genes including ER chaperones and genes encoding the components of ER-associated degradation, such as ERdj4. Upon removal of GRP78, PERK autophosphorylates and activates in ER membrane. Activated PERK phosphorylates and inactivates eIF2α leads to the translational up-regulation of specific mRNAs, such as ATF4. ATF4 is a transcription factor that activates the transcription of pro-apoptotic factors CHOP and GADD34. Release of GRP78 frees ATF6 to translocate to the Golgi apparatus where resident proteases cleave ATF6, releasing this transcription factor into the cytosol and allowing it to migrate into the nucleus and regulate the gene expression [26].

The mechanisms underlying GA-induced ER stress are unknown. In this study, it showed GA could induce XBP1 splicing. However, it is notable that in response to the treatment with tunicamycin the active:inactive XBP1 ratio increased, while in response to the treatment of GA the ratio decreased after 8 h. This was due to cell death induction by GA, resulting in decreased active: inactive XBP1ratio, while tunicamycin did not. Furthermore, GA upregulated the mRNA levels of ER stress-associated molecules, including GRP78, CHOP, ERdj4 and GADD34. GADD34 is a target gene of the UPR and its induction in the early stress response promotes subsequent dephospholylation of eIF2α [27]. Tunicamycin is a typical inducer of ER stress that induces XBP1 splicing and activation of IRE1 [28]. Interestingly, GADD34 expression induced by GA was high, while it was not induced by tunicamycin. This finding suggests that GA induce ER stress in HeLa cells with different mechanism from that observed for tunicamycin. The modulation in the status of GRP-78 differs in response to the treatment with TM and GA could be explained according to Shinjo et al. [29]. The expression patterns of nine UTP target genes induced by seven UTP-inducing compounds including tunicamycin in HeLa cells were reported. These compounds were classified by hierarchical clustering analysis into two clusters; cluster A (thapsigargin, tunicamycin, 2-deoxyglucose, and dithiothreitol) and cluster B (brefeldin A, monensin, and eeyarestatin I). Their study also showed that the expression of GRP78 induced by brefeldin A is lower than by tunicamycin. Therefore, the difference in expression of UPR target gene profiles depends on the mode of action of compounds.

Furthermore, previous studies about ER stress in cancer showed that GRP78 promoted cancer cell proliferation, and not only protects cancer cells from the impact of microenvironment but also provides chemoresistance. Knockdown of GRP78 can suppress cancer cell growth and increase the sensitivity of cancer cells to chemotherapeutic agents [30]. On the other hand, CHOP acts as an antagonist of GRP78 [31]. CHOP induces proapoptotic effect when ER stress is too severe to maintain cellular homeostasis. CHOP stimulates expression of GADD34 resulting in dephosphorylation of eIF2α and recovery of protein translation. High rates of protein synthesis during ER stress lead to cell death through the accumulation of misfolded proteins, which would reduce tumor mass. Then CHOP and GADD34 induce oxidative protein folding. Induction of GADD34 can contribute to chronic stress resulting in exaggerated toxicity and cell death. While suppression of CHOP and GADD34 promoted tumor growth, invasiveness and angiogenesis [32]. Therefore, GA showed little expression of GRP78 but high expression of CHOP and GADD34 may mediate tumor-suppressive effects.

Previous studies showed that GA could induce apoptosis in many cancer cells [2-5]. Liu et al. [33,34] reported that GA alone did not increase expression of CHOP and GRP78 but the combination of GA and calcium channel blocker verapamil or proteasome inhibitors bortezomib, induced high expression of these two proteins especially CHOP protein in human hepatoma HepG2 and leukemia K562 cells. Interestingly, GA alone did not induce apoptosis in both cell types but the combination of GA and verapamil or GA and bortezomib did, which is different from our results that only GA could induce ER stress and apoptosis in HeLa cells. Thus, these results show GA could induce different mechanism of ER stress and apoptosis pathways depending on cancer cell type.

This study also showed that GA induced apoptosis in HeLa cells via caspase-3, −8, −9 and PARP. ER stress can also activate well-known general regulators of apoptosis, including caspases and the Bcl-2 family of proteins. It has been known that Bcl-2 family members reside in the ER membrane and function principally at the mitochondrial outer membrane, so they may influence homeostasis and apoptosis from the ER as well [35,36]. Anti-apoptotic Bcl-2 or Bcl-X_L_ ,targeted specifically to the ER membrane, can block apoptosis induction by pharmacological kinase inhibition or by pro-apoptotic Bcl-2 family members. On the other hand, ER stress can activate several BH3-only pro-apoptotic members of the Bcl-2 family, including Bim, Bik, and Puma [37]. CHOP has been shown to promote apoptotic pathways, downstream of ER stress, by transcriptionally down-regulated the anti-apoptotic protein BCl-2 and up-regulate DR5, a member of the death receptor protein family [20]. We showed that GA could induce both intrinsic and extrinsic apoptotic pathways which involved in down-regulation of Bcl-2 protein, up-regulation of Bim protein as well as activation of caspase-8 and −9 and DR5.

In mammals, MAPKs guide cellular maturation and can induce inflammation and apoptosis. The MAPK family includes ERKs, which are activated by mitogens, and JNKs and p38 MAPKs that primarily activated by cytokines and in response to cellular stress [38]. It has been reported that ER stress caused the activation of JNK signaling pathway through IRE1-TRAF2 (TNF receptor associated factor 2) -ASK1 (Apoptosis signal-regulating kinase 1) pathway and up-regulated the pro-apoptotic activity of DR5, Puma, and Bim leading to apoptosis induction [39,40]. In this study, GA was shown to up-regulate phosphorylated JNK after 8 h of treatment. Furthermore, GA also showed down-regulated phosphorylated ERK but not p38. Our result suggests that GA induces apoptosis via up-regulation of JNK and down-regulation of ERK signaling pathway.

## Conclusion

Although cell death mechanism during ER stress remains unclear, it seems that the death pathways depend both on the cancer type and on the tumor microenvironment. But in this study we have demonstrated for the first time that GA induces apoptosis associated with the ER stress response through up-regulation of JNK and down-regulation of ERK in HeLa cells. This finding suggests that GA could be a potential anticancer agent and these may offer further possibilities for alternative treatment.
